# Unifoliolate Leaves Are Compound in Neotropical Rutaceae: Novel Evidence from Lateral Leaflet Traces

**DOI:** 10.3390/plants15111725

**Published:** 2026-06-02

**Authors:** Carlos Eduardo Valério Raymundo, José Rubens Pirani, Gladys Flávia de Albuquerque Melo-de-Pinna

**Affiliations:** Departamento de Botânica, Instituto de Biociências, Universidade de São Paulo, Rua do Matão 277, São Paulo 05508-090, SP, Brazil; pirani@sp.br (J.R.P.); gfmpinna@usp.br (G.F.d.A.M.-d.-P.)

**Keywords:** Galipeinae, leaf ontogeny, plexus vascular, swollen, unifoliolate, vascularization

## Abstract

Unifoliolate compound leaves have a single leaflet and are common in Galipeinae, the most diverse lineage of Neotropical Rutaceae. In Galipeine, the apical end of the petiole is swollen (apical swollen) has been interpreted as evidence of a compound nature. Additionally, recent studies have also identified traces of lateral leaflets that undergo early abortion. Here, we analyzed leaf ontogeny and vascularization in nine genera to investigate the structural organization and possible compound origin of unifoliolate leaves in Galipeinae. Shoot apices and mature leaves were examined using light microscopy and scanning electron microscopy, and selected species were examined using micro–computed tomography, providing a broader understanding of the vascular architecture in the apical swelling. Two vascularization patterns were identified at the apical swollen: (Type 1) vascular plexus formed by traces of early aborted lateral leaflets (*Erythrochiton brasiliensis* and *Conchocarpus macrophyllus* and others species); (Type 2) a closed vascular (*C. ruber*, *C. fontanesianus* and *C. albiflorus*). The leaflet traces present in type 1 provide novel anatomical evidence of an ancestral compound condition, whereas type 2 suggests simple leaf origins. Our results indicate that swelling apical alone may not be a definitive criterion for classifying leaves as a unifoliolate compound in Galipeinae.

## 1. Introduction

**Figure 1:**
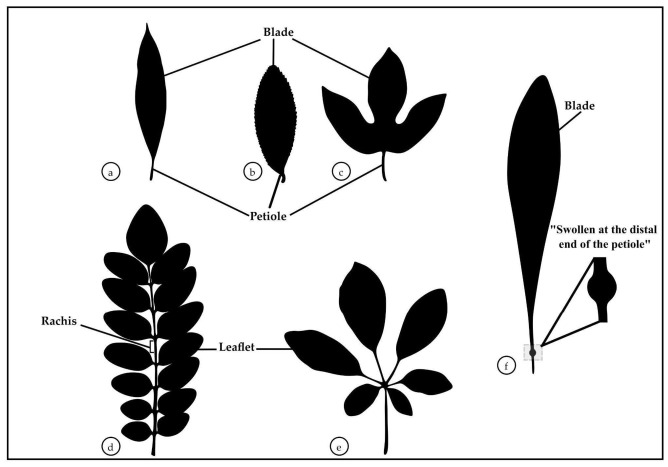
Leaf architecture in angiosperms, based on divisions of the blades. (**a**–**c**) Simple. (**d**) Pinnate, compound. (**e**) Palmate, compound. (**f**) Unifoliolate compound. (**a**–**d**) Simple leaves with entire (**a**) serrated (**b**) and lobed (**c**) margins. (**d**) Pinnate compound leaf with leaflets along the main axis (rachis). (**e**) Palmate compound leaf with leaflets at a single point at the distal end of the petiole. (**f**) Unifoliolate leaf with a swollen at the distal end of the petiole (dashed region).

Angiosperm leaves are appendicular, asymmetrical, and vascular structures originating from the shoot apical meristem (SAM) [1], with remarkable morphological diversity [2], and are classified as simple or compound based on divisions of the leaf blade [3,4,5,6,7,8,9]. Simple leaves have a single, undivided, leaf blade, which can be lobed, serrated, or smooth (Figure 1a–c). Compound leaves are divided into leaflets that vary in number and arrangement (Figure 1d,e). Pinnate compound leaves have more than three leaflets along the main axis (rachis, Figure 1d), while palmate compound leaves have leaflets emerging from a common point at the distal end of the petiole (Figure 1e). A unifoliolate compound leaf is a special case, in which a compound leaf has a single leaflet [10,11] (Figure 1f). Although morphologically similar to simple leaves, unifoliolate leaves differ by the presence of an articulation between the leaf blade and the petiole or a swollen at the distal end of the petiole (Figure 1f), which corresponds to the insertion site of lateral leaflets lost during evolution [10,11,12].

Leaflet reduction in number in a compound leaf can occur evolutionarily due to leaflet loss, fusion of leaflets, or both [13]. Anatomical vascular structure is important for interpreting vestigial structures and their origins [13]. Vascular traces of lost organs often remain in the floral receptacle, even in the absence of the organ itself [13]. Vascular bundles, however, only form when an organ is initiated, and if the organ is absent, its corresponding vascular bundle will not develop [14]. The evolutionary loss of vascular bundle should occur simultaneously with or before the complete disappearance of the organ, implying that the absence of vascular traces is strong evidence of the absence of the organ [14].

Unifoliolate compound leaves are found in several families of angiosperms, including Zygophyllaceae [12], Fabaceae [15], Berberidaceae [16], Oleaceae [17], and particularly in members of the order Sapindales (Sapindaceae, Simaroubaceae, Burseraceae, and Rutaceae [10]. Compound leaves are ancestral in the Rutaceae, the largest family within Sapindales [18]. Evolutionary evidence suggests that reversion to the unifoliolate/simple condition and subsequent return to compound leaves has occurred in some lineages [18].

The unifoliolate leaf typical of most species of *Citrus* (subfamily Aurantioideae, Rutaceae) is the terminal leaflet of an incompletely developed pinnate leaf typical of Sapindales [19]. Unifoliolate leaves are also found in several genera of Aurantioideae [20], a mostly Australasian group, and in the mostly pantropical subfamily Zanthoxyloideae. Unifoliolate leaves in Aurantioideae usually have an, often winged, articulation at the junction of the blade with the petiole.

In Zanthoxyloideae, unifoliolate leaves may be articulated (e.g., *Vepris*, [21], and *Esenbeckia*, [22], with winged or unwinged petioles), but more often are unarticulated with the petiole distally swollen, as observed in some of the neotropical subtribe Galipeinae, tribe Galipeeae. Distal, pulvinus-like thickening, has been suggested as the descriptor for the thickened region near the petiole apex of several Rutaceae [23]. Unifoliolate leaves of two species of *Simaba* (Simaroubaceae) have also been described as bearing a “petiole pulvinate at apex” [24]. In Burseraceae, the petiole is usually described as “often basally pulvinate”, and most species of *Dacryodes*, *Protium*, and *Trattinnickia* have a pulvinulus at least at the distal end of the terminal petiolule and usually at both ends of the lateral petiolules” [25]. Leaves in this family are “imparipinnately compound or infrequently unifoliolate or apparently simple, rarely bipinnate” [25]. Thickening (pulvinus) of petiole is not reported in species in three other large families of Sapindales (Anacardiaceae, Meliaceae, and Sapindaceae), having mostly pinnate leaves, and less often unifoliolate and simple leaves [26,27,28].

While unifoliolate leaves are common in a variety of plants, little is known about this leaf form or its ontogeny. Comparative analysis in the Pilocarpinae (Rutaceae) suggests two (early and late) origins for unifoliolate leaves [29]. The early type is structurally homologous with simple leaves, wherein lateral leaflet primordia are not produced, and lateral leaflets are aborted during the earliest stages of development [29,30]. We observed traces of lateral leaflets in the apical swelling of unifoliolate leaves in the region with leaflet insertion in trifoliolate leaves, suggesting a common origin and indicating an early structural disruption in the development of lateral leaflets [30].

Galipeinae (tribe Galipeeae, Zanthoxyloideae, Rutaceae) is monophyletic [31,32] and the most diverse group of neotropical Rutaceae [33]. Many genera in this subtribe have unifoliolate leaves associated with apical swelling [10,34], making Galipeinae an excellent model for investigation of the evolution and development of leaf morphology. In addition, the group exhibits remarkable diversity in leaf architecture including simple, trifoliolate, and palmate compound forms [34], providing a valuable comparative framework for studies of leaf pattern diversification. This broad morphological variation suggests that Galipeinae may preserve important ontogenetic and anatomical evidence for understanding the origin and evolution of unifoliolate leaves.

Here, we analyzed leaf development with different patterns of division including a single and continuous blade to leaves with three or more leaflets (Table 1), using classical histological techniques and micro–computed tomography (micro-CT), to understand the vascular architecture of the swelling at the distal end of the petiole. We hypothesize that the unifoliolate leaves of Galipeinae originated through the evolutionary reduction and early development abortion of lateral leaflets from ancestrally compound leaves, while preserving vestigial vascular traces within the swollen apical region of the petiole. Our goals were to answer the following questions to understand whether, or in what groups, unifoliolate leaves are compound in origin in Zanthoxyloideae. In the Galipeinae, do unifoliolate leaves follow the same developmental pattern as those previously described for unifoliolate leaves in the Pilocarpinae? Are traces of lateral leaflets a common feature across unifoliolate leaves in Galipeinae, as observed in the unifoliolate condition of *Conchocarpus heterophyllus*? In answering these questions, we attempt to better understand the nature of unifoliolate leaves in Neotropical Rutaceae.

## 2. Results

### 2.1. Ontogeny of Leaf Patterns

**Figure 2:**
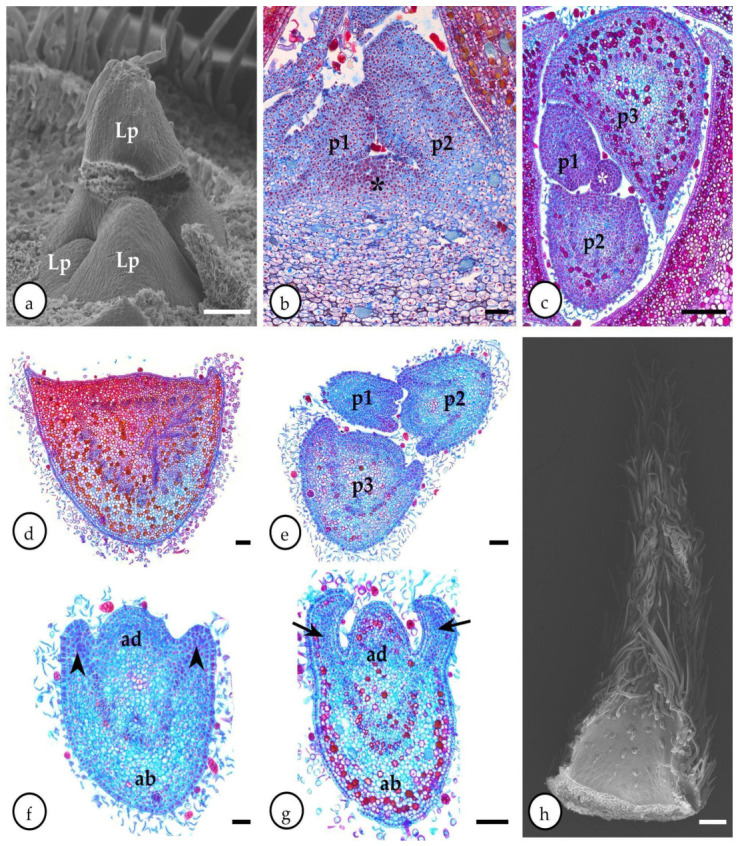
Overview of leaf development in *Erythrochiton brasiliensis*. (**a**) Leaf primordia protecting the SAM and the most recent primordia. (**b**) Longitudinal view of the shoot apex (*) with two leaf primordia. (**c**) Transverse section of the shoot apex (*) with leaf primordia at different differentiation stages. (**d**) Vascular system disorganization, forming a vascular plexus. (**e**) Leaf primordia in the leaf blade domain. (**f**,**g**) Transverse section of the leaf primordium showing the establishment to the marginal meristem between the adaxial/abaxial domains, expanding the leaf blade. Note the differentiation of the procambium (arrow). (**h**) Fully developed unifoliolate leaf primordium. ab = abaxial; ad = adaxial; Lp = leaf primordia; p = plastocrono. Scale bars: (**a**,**c**–**e**,**g**) = 100 μm; (**b**,**f**) = 50 μm; (**h**) = 200 μm.

Unifoliolate leaf development characteristics are consistently shared among the species examined (*Conchocarpus macrophyllus*, *C. macrocarpus*, *C. fontanesianus*, *C. ruber*, *Erythrochiton brasiliensis*, *Neoraputia alba*; Figure 2 and Supplementary Figure S1). Leaf primordia arise from the peripheral zone of the shoot apex and establish the adaxial–abaxial polarity axis (Figure 2a,b). This polarity is histologically observed beginning with the earliest plastochrons (p), as seen by the structural differentiation of tissues in the abaxial domain of the leaf primordium, characterized by vacuolated cells (Figure 2c). The numerous vascular bundles scattered throughout the cortical region in the p5 leaf primordium of *E. brasiliensis*, form a vascular plexus (Figure 2d), not observed in *Conchocarpus ruber.* At this stage, there are no signs of lateral primordia formation (Figure 2d). As the leaf primordia develop, the blade expands laterally along the medio–lateral axis at the intersection of the adaxial/abaxial domains, through the activity of the marginal meristem (Mm; Figure 2e–g and Supplementary Figure S1c–f). At this point, the procambial strand of the blade connects with the procambial center of the leaf rib (Figure 2g). As a result, the basic structure of the unifoliolate leaf is established, consisting of a petiole and an undivided leaf blade (Figure 2h).

**Figure 3:**
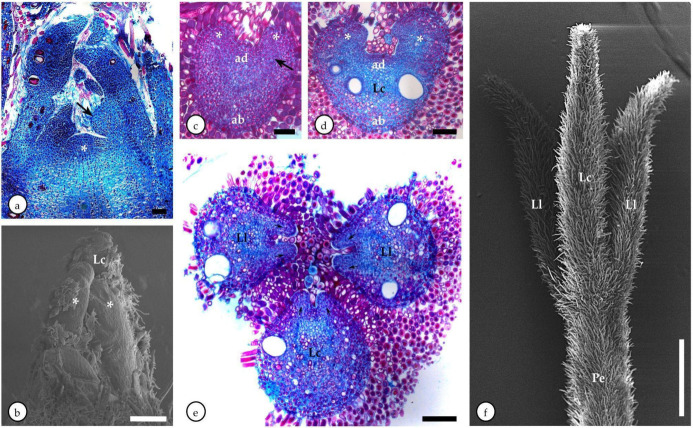
Overview of leaf development in compound leaves of Galipeinae. (**a**) *Galipea laxiflora*. (**b**) *Angostura bracteata*. (**c**–**f**) *Spiranthera atlantica*. (**a**) Longitudinal view of the shoot apex (*) showing a lateral leaflet primordium (arrow). (**b**) Central leaf primordium with a pair of lateral leaflets (*). (**a**–**e**) Transverse sections of different stages of leaflet differentiation. (**c**,**d**) Pair of lateral leaflet primordia positioned between the adaxial and abaxial domains (*). The traces of the lateral leaflet primordia establish a connection with the procambium of the main primordium (arrow). (**e**) Individualized leaflet primordia, with blade expansion in each leaflet, resembling the unifoliolate leaf structure. (**f**) Fully developed trifoliolate compound leaf primordium. ab = abaxial; ad = adaxial; Lc = leaflet central; Ll = lateral leaflet; Pe = petiole. Scale bars: (**a**,**c**–**e**) = 50 μm; (**b**) = 100 μm; (**f**) = 1 mm.

The development of compound leaves with more than one leaflet (as in *Rauia nodosa*, *Angostura bracteata*, and *Spiranthera atlantica*) differs from that of unifoliolate leaves (Figure 3). Leaf primordia are initiated on the flanks of a SAM (Figure 3a). In this leaf pattern, during the early stages of development, each leaf primordium initiates lateral leaflet primordia between its adaxial and abaxial flanks (Figure 3b,c). These leaflets appear as a pair of protrusions emerging from the main primordium (Figure 3c,d). At this stage, the vascular traces of the lateral leaflet primordia establish a connection with the procambium of the main primordium (Figure 3c). At later stages, the leaflet primordia become individualized (Figure 3e), and each reiterates a developmental pattern similar to that of unifoliolate leaves (Figure 3e). In each lateral leaflet primordium, the Mm is established at the adaxial/abaxial interface, promoting the expansion of the leaf blade in the medio-lateral direction (Figure 3e). By the end of development, the basic architecture of the primordium is fully established, comprising three distinct subregions: the petiole, the petiolule, and the leaflet blade (Figure 3f).

### 2.2. Morphology and Vascular Architecture of the Petiole in Mature Leaves

**Figure 4:**
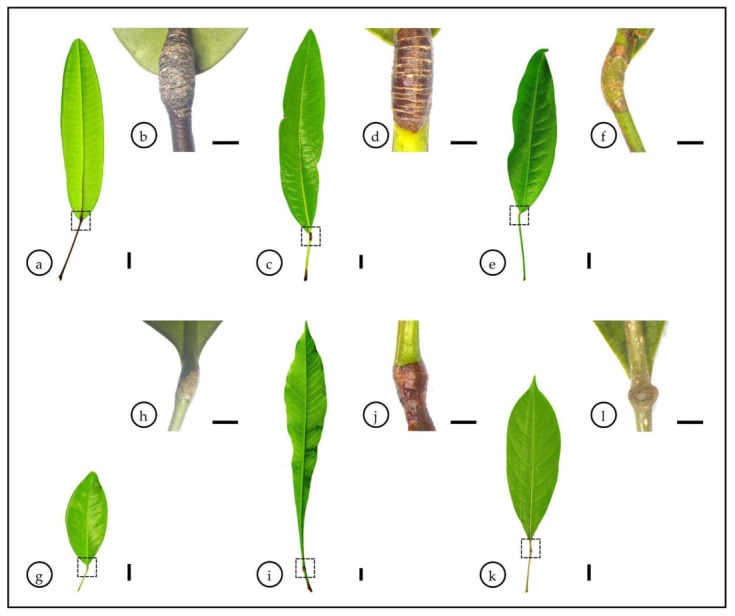
Morphology patterns of unifoliolate leaves in Galipeinae. (**a**,**b**) *Conchocarpus macrophyllus*. (**c**,**d**) *Conchocarpus macrocarpus*. (**e**,**f**) *Conchocarpus ruber*. (**g**,**h**) *Conchocarpus minutiflorus*. (**i**,**j**) *Erythrochiton brasiliensis*. (**k**,**l**) *Neoraputia alba*. (**a**,**c**,**e**,**g**,**i**,**k**) Unifoliolate compound leaves forming a single leaf blade with a swollen apical region (dashed area). (**b**,**d**,**f**,**h**,**j,l**) Detail of the swollen at the distal end of the petiole. Scale bars: (**a**,**c**,**e**,**g**,**i**,**k**) = 2 cm; (**b**,**d**,**f**,**h**,**j,l**) = 2 mm. (Photos. (**a**,**c**,**e**,**g**,**i**,**k**): Gomes Santos L.F).

Scars from reduced lateral leaflets were absent in all unifoliolate leaves examined (Figure 4). A distinctive feature of these leaves is the swollen area between the base of the lamina and the distal end of the petiole (Figure 4a–l). The swollen area is morphologically visible as an increase in diameter compared to the proximal region of the petiole (Figure 4b,d,f,h,j,l).

**Figure 5:**
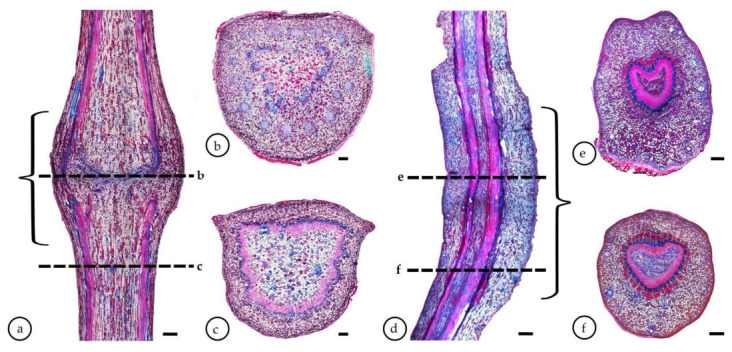
Vascularization pattern of the petiole of Galipeinae. (**a**–**c**) *Erythrochiton brasiliensis*. (**d**–**f**) *Conchocarpus ruber*. (**a**) General view of the petiole vascularization from the swollen apical region and the lower region. (**b**) Vascular plexus formed by traces of lateral leaflets. (**c**) Closed vascular cylinder. (**d**) Closed vascular cylinder, continuous along the petiole. (**e**) Swollen apical region with a closed vascular cylinder. Note the increase in diameter compared to the region below the swollen (**f**). Scale bars: (**a**,**d**) = 500 μm; (**b**,**c**,**e**,**f**) = 200 μm.

**Figure 6:**
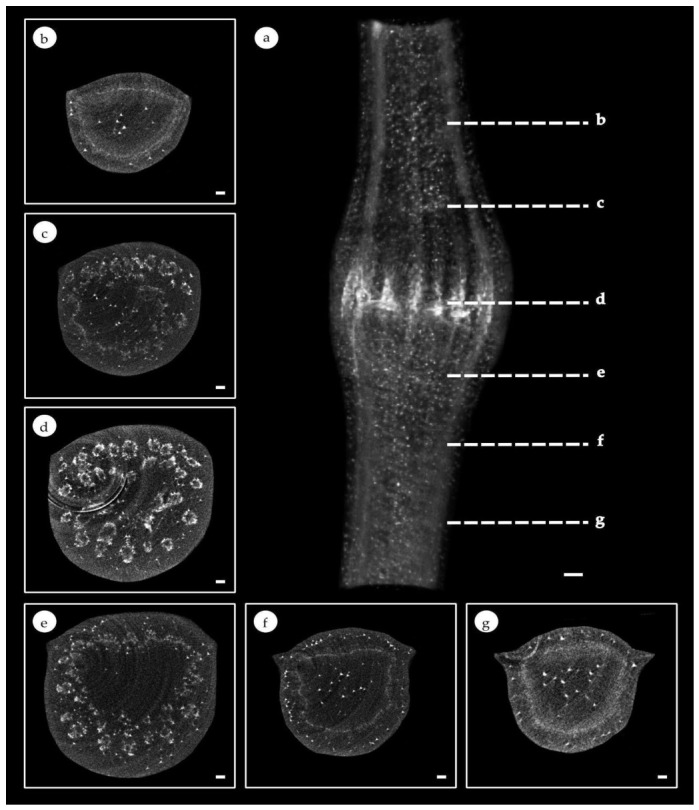
Micro–computed tomography images of *Erythrochiton brasiliensis*. (**a**) General view of the petiole showing trajectory of vascularization. (**b**–**g**) Sequential organization of the vascular system in the transverse section corresponding to regions indicated in (**a**). (**b**,**c**) Region above the swollen area with a closed vascular cylinder and no vascular plexus. (**d**,**e**) Swollen region, showing the lateral leaflet traces. (**f**,**g**) Region below the swollen area with a closed vascular cylinder similar to the region above the swollen area. Scale bars: (**a**) = 100 μm; (**b**–**g**) = 200 μm.

**Figure 7:**
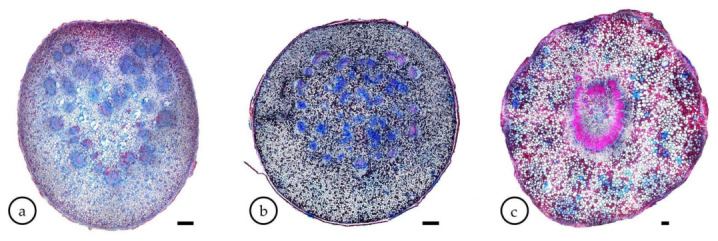
The vascularization pattern in the swollen at the distal end of the petiole of unifoliolate leaves of Galipeinae. (**a**) *Neoraputia alba*. (**b**) *Conchocarpus macrophyllus*. (**c**) *Conchocarpus fontanesianus*. (**a**,**b**) Vascular plexus in the swollen apical region formed by lateral leaflet traces. (**c**) Absence of vascular plexus, with a closed vascular cylinder. Scale bars: (**a**,**b**) = 200 μm; (**c**) = 100 μm.

**Figure 8:**
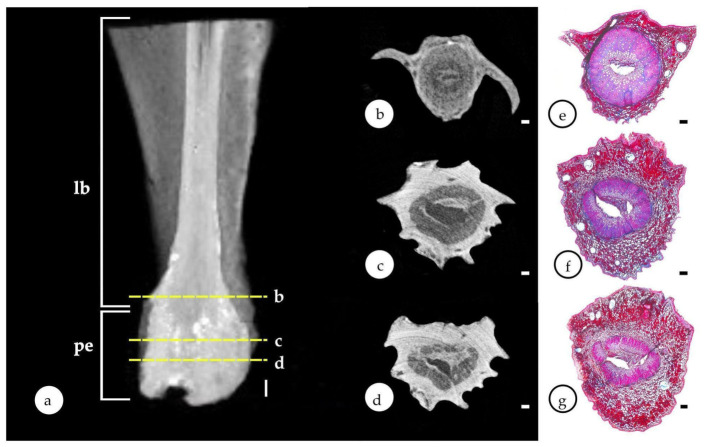
Micro–computed tomography images and anatomical characterization of petiole in *Hortia oreadica*. (**a**) General view of the petiole. (**b**–**d**) Showing the vascular cylinder closed along the petiole. (**e**–**g**) Sections transversal showing the vascular cylinder closed along the petiole. lb = leaf blade; pe = petiole. Scale bars: (**a**) = 100 μm; (**b**–**g**) = 200 μm.

Differences in the vascular system organization are seen in the serial transverse and longitudinal sections, and micro–CT analyses along the petiole axis. In unifoliolate leaves, the vascular system in the distal region of the petiole consists of a closed vascular cylinder (Figure 5a,d and Figure 6a,b and Supplementary Figure S2a–g). Beginning at the swollen region and continuing onwards several bundles form a vascular plexus (Figure 5b, Figure 6c,d and Figure 7a,b). Just below the swollen region, the vascular system of *Conchocarpus macrocarpus*, *C. macrophyllus*, *Neoraputia alba*, and *Erythrochiton brasiliensis* reorganizes, returning to the conformation of a closed cylinder (Figure 5c and Figure 6e–g). However, in *Conchocarpus ruber* (Figure 5d–f), *C. fontanesianus* (Figure 7c) and *C. albiflorus* (Supplementary Figure S2a–g), all described as unifoliolate, the vascular cylinder remains closed and uninterrupted throughout the swollen region. Similarly, in *Hortia oreadica*, the vascular pattern of the petiole in the simple leaf maintains a continuous closed vascular cylinder throughout its entire axis (Figure 8a–g).

**Figure 9:**
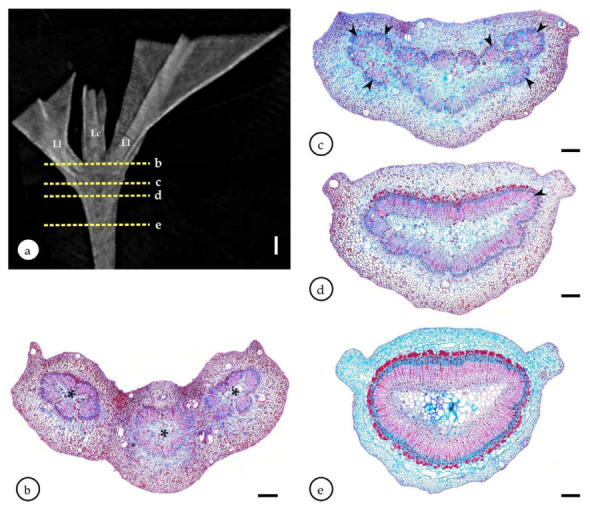
Section sequentially showing the vascularization at the insertion point of the lateral leaflets in the trifoliolate pattern of *Galipea jasminiflora*. (**a**) General view of the petiole of the trifoliolate compound leaf in micro–CT. (**b**–**e**) Transverse section from the distal to the proximal of petiole. (**b**) Insertion point of the three leaflets (*). (**c**,**d**) Traces of the lateral leaflets integrating into the axial system of the central leaflet (arrows). (**e**) Vascular system organized into a closed vascular cylinder. Lc = leaflet central; Ll = lateral leaflets. Scale bars: (**a**) = 100 μm; (**b**–**e**) = 200 μm.

**Figure 10:**
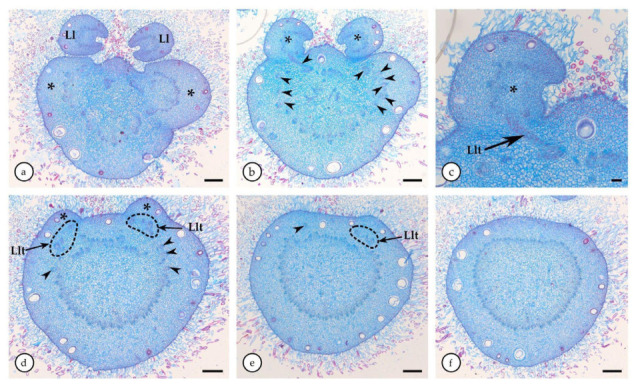
Transverse sections sequentially showing the vascularization at the insertion point of the lateral leaflets in a palmate pattern of *Conchocarpus heterophyllus*. (**a,b**) Insertion point of the lateral leaflets (*), with numerous traces (arrows) forming a vascular plexus. (**c**) Detail of a lateral leaflet vascular trace connecting to the axial system of the central leaflet. (**d**,**e**) Traces of the lateral leaflets integrating into the axial system of the central leaflet. (**f**) Closed vascular cylinder below the insertion point of the lateral leaflets. Ll = lateral leaflet; Llt = traces lateral leaflets. Scale bars: (**a**,**b**,**d**–**f**) = 200 μm; (**c**) = 100 μm.

In trifoliolate and palmate leaves (Figure 9 and Figure 10), from the point of insertion of the lateral leaflets towards the proximal region of the petiole, the vascular connection between the lateral leaflets and the central leaflet is visible (Figure 9a–d and Figure 10a–e). In the insertion region, the vascular system is formed by numerous traces from the lateral leaflets, which together constitute a vascular plexus (Figure 9b,c and Figure 10b). At a certain point, the traces of the leaflets merge with the axial axis of the central leaflet (Figure 9d and Figure 10d,e). Finally, in the proximal region of the petiole, these traces completely merge with the vascular system of the central leaflet, forming a fully closed vascular cylinder (Figure 9e and Figure 10f).

## 3. Discussion

### 3.1. Lateral Leaflet Traces: Anatomical Record of the Compound Identity of Unifoliolate Leaves

Two different patterns of vascular architecture identified in the swollen at the distal end of the petiole appear to correspond to distinct evolutionary trajectories of unifoliolate leaves in Galipeinae: Type 1, vascular plexus, characterized by interconnected vascular bundles associated with lateral leaflet traces, is interpreted as evidence of an ancestral compound developmental program in which lateral leaflets were initiated but early aborted. In contrast, type 2, characterized by a closed vascular cylinder lacking leaflet traces, may represent structurally simple leaves with no evidence of a compound origin. That is, we affirm that, in unifoliolate leaves, evidence in the distal end of petiole provides novel indications of ancestral lateral (compound) leaflets in the neotropical Rutaceae. However, some species with a swollen at the distal end of the petiole lack vascular remnants of ancestral lateral leaflets (Table 2), therefore, a swelling apical alone may not be a definitive criterion for classifying a leaf as a unifoliolate ancestrally compound.

The petiole apex in both palmately and pinnately compound leaves has leaflets that attach to the rachis at a single point or at regular intervals [3,41]. Anatomically, this region has convergent and intermixed vascular bundles at the rachis nodes, analogous to the vascular interactions at the base of the leaf in the nodal region between the leaf and the stem [41]. At each rachis node in pinnate architecture the vascular traces diverge from the main rachis and create a corresponding vascular gap [41]. Terminology of this region is confusing, including “nerving centers of higher degree” [42], “upper vascular plexus” [43] and has not yet been standardized. In palmate forms the region has been called a “plexus of tissues” [44]. We prefer the term “vascular plexus,” as this anatomical configuration is a region of interconnected vascular networks.

Palmately compound leaves have a vascular plexus that is directly associated with leaflet traces. Each leaflet is attached to the central axis of the central leaflet in a region of anastomoses. Anatomically, the vascular plexus in the swollen at the distal end of the petiole of the type 1 unifoliolate leaf has a similar configuration as that of palmately compound leaves. The vascular plexus in unifoliolate leaves comprises vascular bundles associated with lateral leaflet traces that do not develop into leaves, as suggested by the early-unifoliolate-leaves hypothesis [29]. Type 1 unifoliolate leaves are anatomically very similar to those described in *Conchocarpus heterophyllus* [30].

Unifoliolate compound leaves are found in four of nine Sapindales families [10], and in sister-families of the Rutaceae [45], a similar vascular plexus has been described in unifoliolate compound leaves in the genus *Simaba* Aubl. (Simaroubaceae; [46], and the genus *Trichilia* P.Browne (Meliaceae; [47]. Comparable patterns occur in other genera of Rutaceae, including *Esenbeckia* Kunth (J. A. Doe, unpublished manuscript.). Anatomical features in unifoliolate leaves of *Dryades concinna* (Kallunki) Groppo & Kallunki and *Conchocarpus macrophyllus* resemble those we describe here, in a study on swelling at the distal end of the petiole anatomy in four subfamilies of Rutaceae, including members of Galipeinae [23]. Vascular architecture analysis conducted across different lineages may provide further insight regarding the origin of unifoliolate leaves in Rutaceae and Sapindales.

While morphologically similar, the swelling at the distal end of the petiole in type 2 leaves has some differences in comparison with type 1 leaves. In type 2, the vascular cylinder is continuous and does not have insertions of leaflet traces. Thus, in these species the initiation of lateral leaflets does not occur and therefore these leaves should be referred to as simple rather than examples of early unifoliolate leaves (Table 2). In *Esenbeckia leiocarpa* Engl, traditionally known as having simple leaves [22], the swollen region has a closed vascular cylinder [23] that is similar to our type 2.

Conflating type 1 and 2 leaves can result in some taxonomic consequences. For example, there is *Conchocarpus heterophyllus*, sister species to the clade including *C. albiflorus*, *C. ruber*, and *C. coeruleus* [32] that were in the genus *Almeidea* A.St.-Hil. [31,39]. Despite their phylogenetic proximity, only *C. heterophyllus* has traces of lateral leaflets and produces compound leaves with more than one leaflet [30]. While *C. albiflorus* and *C. ruber* have distally swollen petioles, they have simple leaves [39], which is supported by the lack of vascular plexus. This anatomical similarity suggests the same phylogenetic relationship between the species as morphological and molecular data [31,39], and suggests that the ancestral condition of the clade formed by *C. albiflorus*, *C. ruber*, and *C. coeruleus*, includes an evolutionary change that led to the simplification of leaf architecture.

In the Rutaceae, the swellings at the distal end of the petioles are morphologically recognized due to their larger diameter [10,23]. The apically swollen petiole in both type 1 and 2 has a proliferation of cortical tissue, similar to that in *Esenbeckia glandiflora* [29], *Conchocarpus heterophyllus* [30] and others members of Galipeinae and Pilocarpinae [23].

### 3.2. Loss of Morphogenetic Potential: Speculations on the Repression of Leaflets in Unifoliolate Compound Leaves

Studies of unifoliolate leaves previously focused on taxonomy [12,21,48,49], and leaf development in unifoliolate leaves was essentially ignored. Leaf development has been described only recently in the Rutaceae [29,30]. Unifoliolate leaves in Pilocarpinae (Rutaceae) were subdivided into: “Early unifoliolate leaves” and “Late unifoliolate leaves” [29]. Development of unifoliolate leaves examined here in the Galipeinae followed that of “Early unifoliolate leaves,” [29], similar to that described in *Conchocarpus heterophyllus* [30].

**Figure 11:**
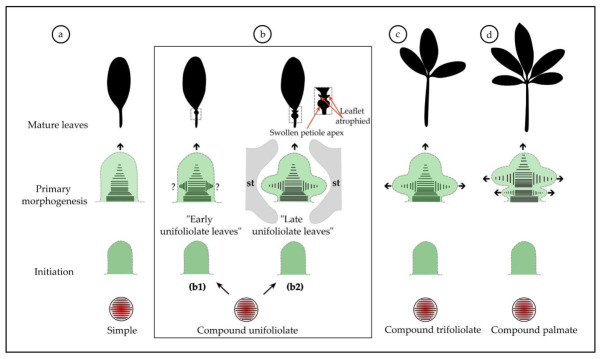
Diagram illustrating hypothesized patterns of leaf development in Rutaceae. (**a**) Simple leaf. (**b**) Unifoliolate compound leaf. (**c**) Trifoliolate compound leaf. (**d**) Palmate compound leaf. All leaf primordia originate from the peripheral zone of the shoot apical meristem (SAM; pink circle). In the initiation phase, a leaf primordium buttress is formed in all leaf types. During primary morphogenesis, the phyllopodium develops and the leaf blade expands through the activity of the marginal blastozon (**a**). In compound leaves (**b**–**d**), the lateral growth of the phyllopodium is subdivided through the initiation of lateral leaflet primordia. Within unifoliolate compound leaves (**b**), two developmental patterns are observed among Rutaceae species: (**b1**) “Early unifoliolate leaves”, with no morphological evidence of lateral leaflet initiation. The factors responsible for the inhibition of these primordia remain unknown (?). (**b2**) “Late unifoliolate leaves”, development is ontogenetically similar to that of a compound leaf. Lateral leaflet primordia are initiated during primary morphogenesis but are later aborted, likely due to spatial constraints imposed by a pair of connate stipules (st), resulting in the formation of atrophied leaflet structures (see Cruz et al., [29]). (**c**) Trifoliolate compound leaves and (**d**) palmate compound leaves exhibit fully developed lateral leaflet primordia.

New structures form from organ primordia and developmental axes become established in morphogenesis primaria [8,9]. In our context, simple leaves grow continuously and result in a single blade (Figure 11a), while compound leaves (Figure 11b–d) have an additional morphogenetic process that leads to the emergence of lateral leaflets from the developing primordia [8,9,50]. While unifoliolate compound leaves appear to be simple leaves, the traces of lateral leaflets suggest that morphogenetic potential in the central leaflet primordium remains. This potential may or may not manifest during unifoliolate leaf development, leading to two different scenarios. The potential does not manifest itself in “Early unifoliolate leaves” and the lateral leaflets are aborted during initiation (Figure 11b). On the other hand, in “Late unifoliolate leaves” this potential manifests, and the lateral leaflet primordia differentiate (Figure 11b), but in this case, space constraints lead to the abortion of the leaflets [29]. In compound leaves (Figure 11c,d), this organogenic phase continues and results in leaves with varying levels of complexity.

Mature leaves in “Early unifoliolate leaves” have a single leaflet, without basal abscission scars. In species with opposite phyllotaxis, such as *Esenbeckia* (Pilocarpinae), the presence of a pair of connate stipules at the leaf base acts as a physical barrier that prevents the development of additional leaflet primordia [29]. The reduction in leaflet number (from three to two, or to a simple leaf) in *Lubaria heterophylla* (Subtribe, Galipeinae, a shrub or treelet with opposite leaves) is suggested to occur through discretionary suppression of leaflet formation or early abortion [49], although this remains untested. In all species studied here, phyllotaxis is alternate and the absence of stipules eliminates this type of mechanical constraint, raising questions about hormonal and molecular mechanisms that may underlie repression of lateral leaflet formation.

At the molecular level, leaf and leaflet initiation are tightly regulated by auxin distribution and transport. Auxin concentrations are at their maximum at the sites of initiation of both leaf and lateral leaflets primordia [50,51,52,53,54,55] and the differentiation of provascular strands [55,56,57], and is evolutionary conserved across angiosperms [53]. Inhibition of auxin transport results in the development of simplified leaves [8,52,53,54,55,56,57]. Chemical inhibitors of auxin transport leads to leaf simplification in tomato (*Solanum lycopersium*) [50,51] while in *Cadarmine hirsuta* it results in simple leaf-like [52]. We hypothesize that auxin signaling in unifoliolate leaves (type 1) was initiated but subsequently interrupted, although it remained sufficient to establish leaflet pre-patterning, including procambial cell differentiation and the formation of vascular traces. This is consistent with the role of auxin in both primordium initiation and vascular system differentiation [55]. Therefore, considering the central role of auxin in vascular identity, future studies in these species will be essential to test the proposed relationship between auxin flux, vascular architecture, and the differentiation of lateral leaflet traces in type 1 unifoliolate leaves of Rutaceae members.

## 4. Materials and Methods

### 4.1. Plant Material

Sixteen species in nine genera (*Angostura*, *Conchocarpus*, *Erythrochiton*, *Galipea*, *Hortia*, *Neoraputia*, *Rauia*, *Ravenia*, and *Spiranthera*; subtribe Galipeinae, tribe Galipeeae, subfamily Zanthoxyloideae, family Rutaceae) were selected (Table 1). Species of *Conchocarpus*, the most diverse genus in the subtribe [58] were prioritized because they exhibit distinct leaf patterns and provide an important comparative framework. Additionally, at least one species from other phylogenetically related genera [31,32] were included for broader comparative analyses.

Most specimens were collected from live plants in the field or cultivated in the living collection of the Instituto de Biociências, Universidade de São Paulo, with voucher specimens deposited in the herbarium of the Universidade de São Paulo (SPF), São Paulo, Brazil (Table 1). We sampled at least three shoot apices and three leaves per specimen of three different individuals per species, which were fixed upon collection in FAA 50 solution (formalin–acetic acid–50% ethanol) for 24 h and subsequently stored in 70% ethanol, following Johansen [59]. Additional samples include dried leaves (three samples per species) of herbarium vouchers (SPF) and rehydrated following Smith and Smith [60] before being stored in 70% ethanol. Samples were analyzed under stereomicroscopy, light microscopy, and scanning electron microscopy (SEM), and, for some samples, high-resolution micro–computed tomography (micro-CT).

### 4.2. Structural Analyses

The overall morphologies of leaf blades and petioles were observed using a Leica M80 stereomicroscope. For anatomical characterization, samples from both fixed material and herbarium specimens (stored in 70% ethanol) were dehydrated through a tertiary butyl alcohol series (50–100%) and embedded in Paraplast, following Johansen [59]. Serial transverse and longitudinal sections (10 μm thick) of the petiole, swollen at the distal end of the petiole, and shoot apex were obtained using a rotary microtome (Leica Microsystems, Wetzlar, Germany). The sections were stained with 1% safranin in 50% ethanol and 1% Astra blue in 50% ethanol, according to Bukatsh’s method [61], and permanently mounted on slides with Entelan. Anatomical analysis was conducted using a Leica DMLB light microscope (Leica Microsystems, Wetzlar, Germany), and samples were digitally photographed for documentation. The terminology used to describe the vascular architecture at the distal end of the petiole following Raymundo et al. [30].

For SEM analyses, shoot apices and petiole (swollen at the distal end of petiole) were dissected, dehydrated through an ethanol gradient series, critical point dried, and then mounted on metal stubs and sputter–coated with gold following Silveira [62]. Samples were then imaged using a Zeiss Sigma VP scanning electron microscope (Carl Zeiss, Oberkochen, Germany).

### 4.3. Micro–Computed Tomography (Micro–CT)

In order to compare vascular architecture, we prioritized representative species encompassing the main anatomical patterns observed in Galipeinae, including taxa with a swollen at the distal end of petiole (*Erythrochiton brasiliensis* and *Conchocarpus albiflorus*) and a species with simple leaves (*Hortia oreadica*) used for structural comparison. Samples of *Erythrochiton brasiliensis*, previously fixed in FAA 50 solution, washed and stored in 70% ethanol [59], and herborized samples of *Hortia oreadica* and *Conchocarpus albiflorus*, rehydrated and preserved in 70% ethanol [60], were used for micro–CT. The sample was wrapped in plastic film and mounted onto the stage of micro–CT equipment, then scanned using a Skyscan 1176 X–ray Microtomograph (Bruker Belgium S.A./N.V., Kontich, Belgium) in the Biosciences Institute, University of São Paulo. This analysis was performed under the following parameters: (voltage (40 kV), current (600 uA), image rotation (−0.3630) and resolution (9 μm). After 3D reconstruction, images were generated with CTVox 3D visualization software version 2.4.0r870. The results obtained with this technique were compared with anatomical sections, offering a more comprehensive understanding of the vascular patterns in leaves.

## 5. Conclusions

Two different vascular architectures in the swollen at the distal end of the petiole suggest that unifoliolate leaves in Galipeinae have followed distinct evolutionary trajectories. In early unifoliolate leaves (e.g., *Conchocarpus macrophyllus*, *C. macrocarpus*, *C. minutiflorus*, *N. alba* and *E. brasiliensis*), the absence of lateral leaflets is associated with the loss of morphogenetical potential in the central leaflet primordium, and is a derived condition that retains remnants of compound leaf development program. In contrast, some species, including *Conchocarpus ruber*, *C. albiflorus*, and *C. fontanesianus* retain no anatomical evidence of leaflet initiation, and have a structurally simple leaf with no indication of a compound origin. In the future, it will be interesting to examine *Conchocarpus* (the most species rich genus in Galipeinae) more thoroughly because some species have lateral leaflet traces while others lack them entirely. It will also be important to investigate whether the lateral leaflet traces observed at the petiole apex are developmentally associated with the formation of the swollen petiole region. Furthermore, in *C. heterophyllus*, the same individual can have both unifoliolate and compound leaves, supporting the hypothesis that the molecular machinery for leaflet formation is present in unifoliolate leaves but repressed early. Future work will be required to understand how these regulatory pathways operate, particularly the dynamics of auxin distribution and its interaction with morphogenetic competence for elucidating the developmental basis of unifoliolate leaves in Rutaceae and across Sapindales.

## Figures and Tables

**Table 1:** Species selected for this study and their collection.

Species	Leaf Characteristics	Voucher and Collection Site	References
*Erythrochiton brasiliensis* Nees & Mart.	Unifoliolate or simple	J.H.L. El Ottra 236 cultivated. IB–USP, São Paulo (SP)	[34,35]
*Galipea laxiflora* Engl.	3–foliolate	J.R. Pirani 6740, Coqueiral, Aracruz, Espírito Santo (ES)	[34]
*Galipea jassminiflora* (A.St.-Hil.) Engl.	3–foliolate	G. Hashimoto s/n (SPF 258475), Ribeirão Preto, São Paulo (SP)	[34]
*Spiranthera atlantica* Pirani	3–foliolate	J.R. Pirani 6732, Reserva Vale, Linhares, Espírito Santo (ES	[34,36]
*Angostura bracteata* (Nees & Mart.)	3–foliolate	J.R. Pirani 6732, Reserva Vale, Linhares, Espírito Santo (ES)	[34,37]
*Rauia nodosa* (Engl.) Kallunki	3–foliolate	J.R. Pirani 6739, Coqueiral, Aracruz, Espírito Santo (ES)	[34]
*Neoraputia alba* (Nees & Mart.) Emmerichex Kallunki	Unifoliolate or simple	C.E.V Raymundo s/n cultivated. IB–USP, São Paulo (SP)	[34,38]
*Conchocarpus macrocarpus* (Engl.) Kallunki & Pirani	Unifoliolate or simple	J.H.L. EL Ottra s/n cultivated. IB–USP, São Paulo (SP)	[34,37]
*Conchocarpus ruber* (A.St.-Hil.)	Unifoliolate or simple	C.E.V Raymundo s/n cultivated. IB–USP, São Paulo (SP)	[34,39]
*Conchocarpus macrophyllus* J.C.Mikan	Unifoliolate or simple	C.E.V Raymundo s/n cultivated. IB–USP, São Paulo (SP)	[34,37]
*Conchocarpus minutiflorus* Groppo & Pirani	Unifoliolate or simple	C.E.V Raymundo s/n cultivated. IB-USP, São Paulo (SP)	[34]
*Conchocarpus fontanesianus* (A.St.-Hil.) Kallunki & Pirani	Unifoliolate or simple	C.E.V Raymundo s/n, Ilhabela, São Paulo (SP)	[34,37]
*Conchocarpus heterophyllus* (A.St.-Hil.) Kallunki & Pirani	Unifoliolate and (2–7)–foliolate	C.E.V Raymundo s/n cultivated. IB–USP, São Paulo (SP)	[30,34,37]
*Conchocarpus albiflorus* (Bruniera & Groppo) Bruniera & Groppo	Unifoliolate or simple	M. Groppo 1861, Cachoeiro de Itapemirim, Espírito Santo (ES)	[31,34]
*Hortia oreadica* Groppo, Kallunki & Pirani	Simple	G. Hashimoto s/n (SPF 258461), Luziânia, Goiás (GO)	[34,40]
*Ravenia infelix* Vell	Simple	J.R. Pirani 6118, Colatina, Espírito Santo (ES)	[34]

**Table 2:** Leaf pattern and vascularization at the apical region of the petiole as defined by the present study.

Species	Leaf Pattern	Swollen Apex	Vascularization	Type
*Erythrochiton brasiliensis*	Unifoliolate	Present	Plexus with vascular bundles of lateral leaflets	Type 1
*Galipea laxiflora*	3–foliolate	Absent	Plexus with vascular bundles of lateral leaflet	Type 1
*Galipea jassminiflora*	3–foliolate	Absent	Plexus with vascular bundles of lateral leaflet	Type 1
*Spiranthera atlantica*	3–foliolate	Absent	Plexus with vascular bundles of lateral leaflet	Type 1
*Angostura bracteata*	3–foliolate	Absent	Plexus with vascular bundles of lateral leaflet	Type 1
*Rauia nodosa*	3–foliolate	Absent	Plexus with vascular bundles of lateral leaflet	Type 1
*Neoraputia alba*	Unifoliolate	Present	Plexus with vascular bundles of lateral leaflet	Type 1
*Conchocarpus macrocarpus*	Unifoliolate	Present	Plexus with vascular bundles of lateral leaflet	Type 1
*Conchocarpus ruber*	Simple	Present	Vascular cylinder	Type 2
*Conchocarpus macrophyllus*	Unifoliolate	Present	Plexus with vascular bundles of lateral leaflet	Type 1
*Conchocarpus minutiflorus*	Unifoliolate	Present	Plexus with vascular bundles of lateral leaflet	Type 1
*Conchocarpus fontanesianus*	Simple	Present	Vascular cylinder	Type 2
*Conchocarpus heterophyllus*	Unifoliolate and (2–7)–foliolate	Present	Plexus with vascular bundles of lateral leaflet	Type 1
*Conchocarpus albiflorus*	Simple	Present	Vascular cylinder	Type 2
*Hortia oreadica*	Simple	Present	Vascular cylinder	Type 2
*Ravenia infelix*	Simple	Absent	Vascular cylinder	Type 2

Type 1 (presence of leaflet traces) and Type 2 absent for traces.

## Data Availability

Data sharing is not applicable to this article.

## Supplementary Materials

The following supporting information can be downloaded at: https://www.mdpi.com/article/10.3390/plants15111725/s1, Figure S1: Leaves with one leaf blade are described as unifoliolate or simple. (**a**,**c**) *Conchocarpus ruber*. (**b**,**d**) *Conchocarpus macrocarpus*. (**e**) *Neoraputia alba*. (**f**) *Conchocarpus macrophyllus*. (**a**) Shoot apex with leaf primordia of *Conchocarpus ruber*. (**b**) Leaf primordial of the *Conchocarpus macrocarpus*. (**c**–**f**) Transverse section of the leaflet showing marginal growth (arrows) at the interface of the adaxial/abaxial domain. ab = abaxial; ad = adaxial; Lp = leaf primordia. Scale bars: (**a**) = 20 μm; (**b**,**c**,**d**) = 100 μm; (**e**,**f**) = 50 μm. Figure S2: Transverse sections sequentially showing the vasculature in *Conchocarpus albiflorus*. (**a**–**d**) Micro-CT. (**e**–**g**) Light microscopy. (**a**) Frontal view along the petiole and leaf blade region, highlighting the continuous vascular cylinder. (**b**–**d**) Transverse view of the vascular architecture. (**e**–**g**) Closed vascular cylinder along the mid-to-upper region of the petiole. Scale bars: (**a**–**d**) = 200 μm; (**e**–**g**) = 100 μm.
