# Supplementary material for: Unifoliolate Leaves Are Compound in Neotropical Rutaceae: Novel Evidence from Lateral Leaflet Traces
Source: Plants (Basel). 2026 Jun 2;15(11):1725. doi: 10.3390/plants15111725 (PMC13259490; doi:10.3390/plants15111725)
Supplement: Supplementary file 1 [file plants-15-01725-s001.zip › plants-4278029-supplementary.pdf]

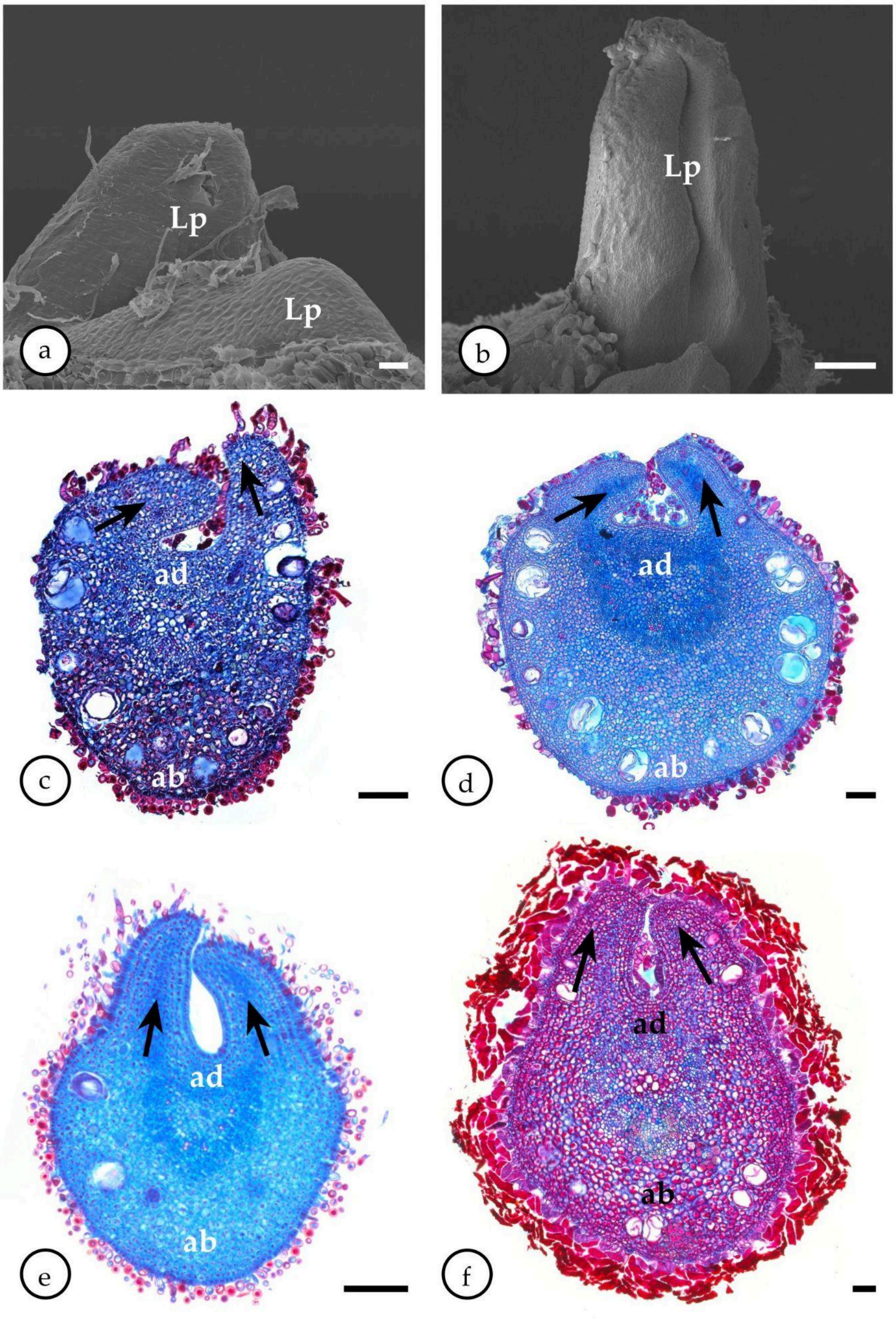

Figure S1: Leaves with one leaf blade are described as unifoliolate or simple. (**a**,**c**) *Conchocarpus ruber*. (**b**,**d**) *Conchocarpus macrocarpus*. (**e**) *Neoraputia alba*. (**f**) *Conchocarpus macrophyllus*. (**a**) Shoot apex with leaf primordia of *Conchocarpus ruber*. (**b**) Leaf primordial of the *Conchocarpus macrocarpus*. (**c**–**f**) Transverse section of the leaflet showing marginal growth (arrows) at the interface of the adaxial/abaxial domain. ab = abaxial; ad = adaxial; Lp = leaf primordia. Scale bars: (**a**) = 20 μm; (**b**,**c**,**d**) = 100 μm; (**e**,**f**) = 50 μm.

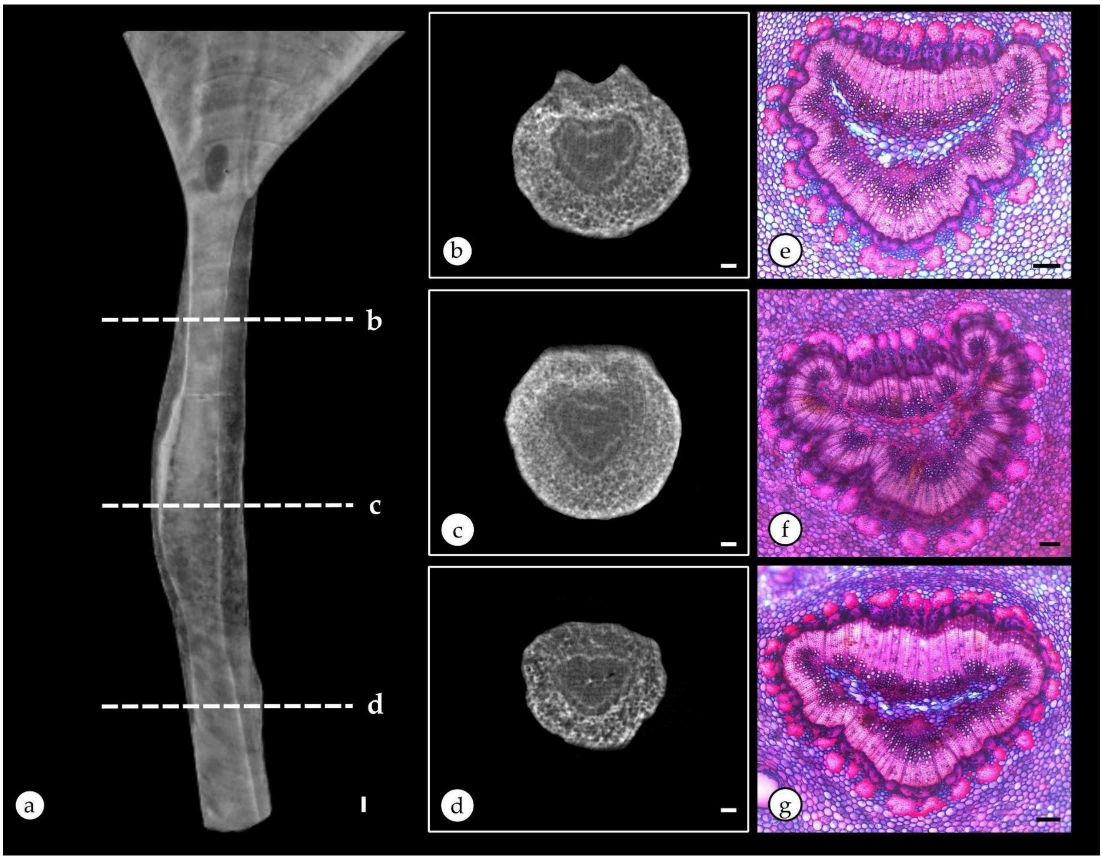

Figure S2: Transverse sections sequentially showing the vasculature in *Conchocarpus albiflorus*. (**a**–**d**) Micro-CT. (**e**–**g**) Light microscopy. (**a**) Frontal view along the petiole and leaf blade region, highlighting the continuous vascular cylinder. (**b**–**d**) Transverse view of the vascular architecture. (**e**–**g**) Closed vascular cylinder along the mid-to-upper region of the petiole. Scale bars: (**a**–**d**) = 200 μm; (**e**–**g**) = 100 μm.
